# Multi-level multi-domain statistical shape model of the subtalar, talonavicular, and calcaneocuboid joints

**DOI:** 10.3389/fbioe.2022.1056536

**Published:** 2022-12-05

**Authors:** Andrew C. Peterson, Rich J. Lisonbee, Nicola Krähenbühl, Charles L. Saltzman, Alexej Barg, Nawazish Khan, Shireen Y. Elhabian, Amy L. Lenz

**Affiliations:** ^1^ Department of Orthopaedics, University of Utah, Salt Lake City, UT, United States; ^2^ University Hospital Basel, Basel, Switzerland; ^3^ University Medical Center Hamburg-Eppendorf, Hamburg, Germany; ^4^ School of Computing, College of Engineering, University of Utah, Salt Lake City, UT, United States; ^5^ Scientific Computing and Imaging Institute, College of Engineering, University of Utah, Salt Lake City, UT, United States; ^6^ Department of Biomedical Engineering, College of Engineering, University of Utah, Salt Lake City, UT, United States; ^7^ Department of Mechanical Engineering, College of Engineering, University of Utah, Salt Lake City, UT, United States

**Keywords:** foot and ankle, statistical shape modeling, computational morphometrics, midtarsal joint locking, weightbearing computed tomography

## Abstract

Traditionally, two-dimensional conventional radiographs have been the primary tool to measure the complex morphology of the foot and ankle. However, the subtalar, talonavicular, and calcaneocuboid joints are challenging to assess due to their bone morphology and locations within the ankle. Weightbearing computed tomography is a novel high-resolution volumetric imaging mechanism that allows detailed generation of 3D bone reconstructions. This study aimed to develop a multi-domain statistical shape model to assess morphologic and alignment variation of the subtalar, talonavicular, and calcaneocuboid joints across an asymptomatic population and calculate 3D joint measurements in a consistent weightbearing position. Specific joint measurements included joint space distance, congruence, and coverage. Noteworthy anatomical variation predominantly included the talus and calcaneus, specifically an inverse relationship regarding talar dome heightening and calcaneal shortening. While there was minimal navicular and cuboid shape variation, there were alignment variations within these joints; the most notable is the rotational aspect about the anterior-posterior axis. This study also found that multi-domain modeling may be able to predict joint space distance measurements within a population. Additionally, variation across a population of these four bones may be driven far more by morphology than by alignment variation based on all three joint measurements. These data are beneficial in furthering our understanding of joint-level morphology and alignment variants to guide advancements in ankle joint pathological care and operative treatments.

## 1 Introduction

Historically, the complex nature of foot and ankle joint morphology has primarily been analyzed individually from two-dimensional (2D) measurements on conventional radiographs (Lopez-Ben, 2015; Krähenbühl et al., 2017). These methods fail to illustrate the complexity of the foot and ankle joints and their spatial relationships. The subtalar, talonavicular, and calcaneocuboid joints are specifically challenging to assess radiographically due to their intricate morphologies and locations within the ankle (Ebraheim et al., 1999; Willauer et al., 2014; Krähenbühl et al., 2017; Bernasconi et al., 2021). Yet, accurately visualizing these three joints is crucial to comprehending the compensatory joint mechanics as well as treatment for multiple pathologies, such as osteoarthritis and progressive collapsing flatfoot deformity (PCFD) (Sammarco, 2004; Bruening et al., 2018; Welte et al., 2021). However, advancements in volumetric imaging, including computed tomography (CT), have made it possible and practical to generate high-resolution three-dimensional (3D) reconstructions of bones throughout the foot and ankle (Hayes et al., 2006; Barg et al., 2018). While these imaging modalities are typically performed in a non-weightbearing position, weightbearing cone-beam CT (WBCT) technology allows for the analysis of joint relationships in a natural and consistent position with the presence of load (Colin et al., 2014; Burssens et al., 2016; Krähenbühl et al., 2016). A better understanding of hind- and midfoot morphometrics could assist in diagnosing and treating multiple joint diseases. However, it can be challenging to quantitatively extract morphological metrics from 3D surface reconstructions while maintaining anatomical relevance.

Statistical shape modeling (SSM) is a population-based mathematical approach to objectively quantify these morphological metrics (Davies et al., 2002; Styner et al., 2003; Cates et al., 2007; Datar et al., 2009; Datar et al., 2011; Goparaju et al., 2022). Using SSM, a statistical model can be created to compare mean bone shape morphology and identify anatomical modes of variation. Previous ankle SSM studies have been limited to a single bone and could not, therefore, evaluate multi-domain joint relationships (Melinska et al., 2015; Melinska et al., 2017; Tümer et al., 2019a; Tümer et al., 2019b; Gabrielli et al., 2020; Krähenbühl et al., 2020; Liu et al., 2020; Schmutz et al., 2021; Arbabi et al., 2022; Peiffer et al., 2022; Vafaeian et al., 2022). A multi-domain technique can be implemented to capture morphological and alignment changes for multiple bones throughout a population. Additionally, multi-level analyses allow for separating morphology and alignment to identify their individual contributions to joint relationships.

Using a multi-domain SSM approach, individual joint-level 3D morphometrics can be calculated to predict variations within the joints based on the original alignment from WBCT scans. These joint metrics include coverage area, joint space distance, and joint congruence. The coverage area is a mathematically derived calculation of the joint’s articulating region. Coverage area is also used to quantify morphology variation, such as osteoarthritis development (Schaefer et al., 2012), and alignment variation, such as joint subluxation (Louie et al., 2014). Calculated from within the joint’s coverage area, the joint space distance is calculated from the Euclidean distance across the joint, and the congruence index rates how well the two articular surfaces match one another (Ateshian et al., 1992; Lenz et al., 2021). Similar to coverage area variations, joint space distance can help clinically indicate degenerative diseases like osteoarthritis (Day et al., 2020) and pathologies with varying alignments, such as PCFD (Bernasconi et al., 2021). These analyses allow for a holistic understanding of bone relationships in the foot and ankle that can guide implant design and development while providing insight into the involvement of multiple joints within various pathological diseases.

This study aims to characterize asymptomatic joint-level morphology and alignment differences throughout statistically significant modes of variation and the joint-level measurements within the subtalar, talonavicular, and calcaneocuboid joints. Additionally, this study aims to determine which, if any, multi-level multi-domain SSM modes of variation that seek to separate morphology from joint alignment can be used to predict joint-level measurements across a population. The validity of the multi-domain SSM approach was assessed by comparing the multi-domain statistical models to models run *via* a single-domain SSM approach. We hypothesized that the first mode of variation could predict joint coverage, distance, and congruence for the population. We also hypothesized that multi-domain SSM does not affect the mean shape compared to single-domain SSM. To accomplish these aims and test these hypotheses, we have presented a new computation approach to evaluate multi-domain influences of morphology and alignment in the case of four bones within the ankle and statistically evaluated the downstream joint measurements.

## 2 Materials and methods

Twenty-seven asymptomatic participants (age: 50.0 ± 7.3 years; height: 169.4 ± 6.4 cm; BMI: 25.3 ± 3.8 kg/m^2^; seven males) were enrolled with IRB approval from an ethics committee (Kantonsspital Baselland, Switzerland; University of Utah). Individuals between 40 and 70 years of age without a history of ankle injury or surgery were considered. Before the study, a clinical and radiographic assessment was performed to exclude participants with a planovalgus or cavovarus deformity.

Each participant underwent a unilateral weightbearing CT (WBCT) scan (Planmed Verity, Planmed Oy, Helsinki, Finland; 0.4 mm isotropic pixel resolution). The WBCT scans were segmented, decimated, and smoothed to generate 3D surface models of the talus, calcaneus, navicular, and cuboid (Amira, v6.0.1, Visage Imaging, San Diego, CA, United States).

### 2.1 Statistical shape modeling

**FIGURE 1:**
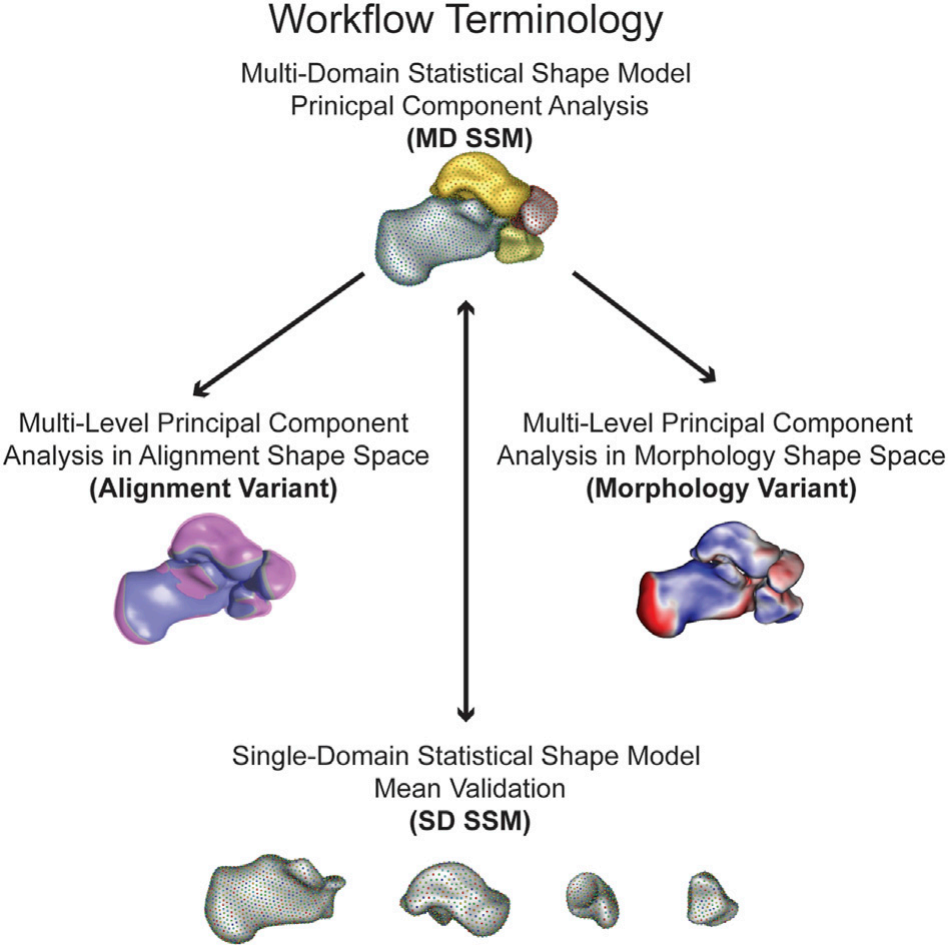
Workflow terminology for the model analysis performed with the simplified terminology bolded. The multi-domain statistical shape model principal component analysis (MD SSM) is a four bone model maintaining anatomical joint relationships analyzing morphology and alignment. The multi-level principal component analysis in alignment shape space (alignment variant) is a four bone model maintaining anatomical joint relationships solely analyzing alignment. The multi-level principal component analysis in morphology shape space (morphology variant) is a four bone model maintaining anatomical joint relationships solely analyzing morphology. The single-domain statistical shape model mean validation (SD SSM) is four single bone models to validate morphology variation to the MD SSM.

For each of the twenty-seven individuals, the talus, calcaneus, navicular, and cuboid were used to create two types of statistical models utilizing an opensource SSM software (ShapeWorks v6.2.1, University of Utah; www.shapeworks.sci.utah.edu). This constructed Particle-based Shape Models (PSM) that automatically placed a dense set of corresponding landmarks on the given set of shapes using an entropy-based optimization scheme (Cates et al., 2017). The SSM approaches included a single-domain SSM (SD SSM) of only the individual bones and a multi-domain SSM (MD SSM) comprised of all four bones in their anatomical alignment (Figure 1). Before optimizing the shape model, any bone models from a left limb were reflected to the right. Using a global iterative closest point algorithm, the input bones for each of the statistical models were aligned. The individual bones were aligned separately for the single-domain model, and each of the four bones were aligned together for the multi-domain model. Aligning the bone models together maintained the individual anatomical alignments from the WBCTs (Wilm, 2022).

The shape model for the four bones is built together by a multi-domain shape modeling approach (Cates et al., 2008), where point correspondences for all the surfaces are optimized in the full joint space of the object complex. A total particle count of 1,024 for the talus, 2,048 for the calcaneus, 512 for the navicular, and 512 for the cuboid was used for both the single and multi-domain statistical models. These correspondence particles were used to define mean shapes and statistically quantify shape differences between bones within the different statistical models. A Procrustes analysis was not applied for these statistical models to remove scale as a factor. Patient specific joint measurements include size variations across the population. In order to compare our population joint level measurements with SSM models, we chose not to use Procrustes for equivalent statistical comparisons. A principal component analysis (PCA) was used to simplify the data to a smaller set of linearly uncorrelated components, or modes of variation. Using a parallel analysis algorithm, statistically significant modes of variation were calculated (*p* < 0.05) (Horn, 1965; Ledesma and Valero-Mora, 2007). For each significant mode of variation, the surface distances between the mean and first and second standard deviation (SD) shapes were calculated and visualized *via* CloudCompare (v2.11. alpha, www.cloudcompare.org). The shape model was analyzed using two different approaches. The first is the multi-domain approach (MD SSM), where PCA analysis is done on the entire multi-bone joint structure treated as a single bone complex reflecting the modes of variation of the entire multi-object complex. The other analysis approach is the multi-level technique, where PCA analysis is done separately on the shape of each individual bone and on the alignment of the entire multi-bone joint structure. This multi-level approach allowed for separately analyzing the alignment shape space model (alignment variant) and the morphology shape space model (morphology variant) (Figure 1). The same significant modes of variation were used for subsequent calculations. For the morphology variant, surface distances between the mean shape and ±1 SD shapes were calculated and visualized *via* CloudCompare, similar to the MD SSM approach. And for the alignment variant, the mean shape and ±1 SD shapes for each mode were overlaid on each other to visualize those variations.

### 2.2 Joint coverage, distance, and congruence index calculations

The correspondence particles from each statistical model were used to automatically calculate and compare the joint-level measurements with an available toolbox (Peterson, 2022). Joint coverage was calculated automatically using normal vectors from the faces of the individual bone models. For a given bone of a joint, if the normal vectors from that bone intersected with a face of the opposing bone, they were considered within the coverage region for that side of the joint. The surface area of the coverage region was then calculated by summing the surface area of the faces that comprised the coverage region. Subject-specific correspondence particles within the coverage region would then be used for that bone’s side of the joint analysis. These identified correspondence particles were paired with their nearest neighboring surface mesh node. It was from this paired node that the joint space distance and congruence index calculations were made. This was necessary as calculating the congruence index requires the mean and Gaussian curvatures of the two opposing surfaces. The joint space distance at a correspondence particle was calculated as the Euclidean distance between its paired node and the nearest opposing surface node. The congruence index for that correspondence particle was then calculated between these two nodes. Congruence index, first described by Ateshian *et al*., is a rating of how well two surfaces match one another, with a congruence index of 0 mm^-1^ being rated as perfectly congruent (Ateshian et al., 1992; Lenz et al., 2021). Both results were mapped to that specific correspondence particle. For visualization, correspondence particles with a distance value greater than 6 mm were colored white for both joint space distance and congruence index. Subsequent statistics were performed on all correspondence particles within the coverage regions

### 2.3 Population vs. PCA modal comparison

The mean and ± 1 SD for coverage across the twenty-seven participants for all four joints were automatically calculated. These average values will be referred to as population calculations throughout this study. Due to the nature of correspondence particles, each distance value and congruence index were paired with identical correspondence particles across the entire population. Thus, the mean and ± 1 SD for distance and congruence can be calculated at each correspondence particle

These calculations for each measurement across the population were statistically compared to the mean and ±1 SD for each significant mode of variation using an unpaired *t*-test based on the means, SDs, and population size. This statistical comparison was also used to compare the alignment and morphology of multi-level multi-domains to the population. A *p*-value less than 0.05 indicates that the two groups are significantly different from one another.

## 3 Results

### 3.1 Statistical shape model

Statistical analysis is performed using Principal Component Analysis (PCA) for the MD SSM, morphology variant and alignment variant, where the mean shape and modes of variation are computed based on the optimized MD SSM (Figure 1). This study observed three statistically significant PCA modes of variation, accounting for 74.8% of the overall shape variation. The three modes represented 63.8%, 6.3%, and 4.7% of the variation. Additionally, when comparing the SD SSM of each bone to the MD SSM, there were negligible differences (<0.1 mm) in mean shape using the previously mentioned surface distance calculations in CloudCompare. Computationally, this statistical shape model took about 40 min to run optimization and the joint measurement analysis took an average of about 3 min to run per patient for the largest joint.

#### 3.1.1 Multi-domain statistical shape model analysis (MD SSM)

**FIGURE 2:**
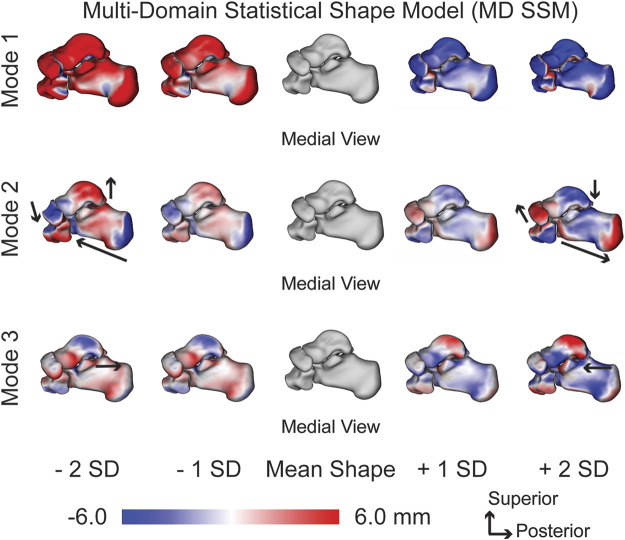
MD SSM modes of variation one to three showing both morphological and alignment significant variations at ± 1 and 2 standard deviations from the mean shape. Black arrows highlight key anatomical feature variations. Red regions are larger than the mean shape and blue regions are smaller than the mean shape with a scale in millimeters (mm).

Substantial morphological and configurational variations were observed while maintaining joint articular relationships within the MD SSM approach. The first PCA mode of variation highlighted size variation primarily, with overall growth and shrinkage of all four bones simultaneously (Figure 2). The second PCA mode is multifaceted in the presented variation. The primary regions of change occur in the inverse relationship between the talus and calcaneus. Concerning the talus, as the talar dome heightens, the posterior process diminishes. Concerning the calcaneus, as the calcaneus lengthens, the posterior facet’s slope decreases. Analyzing the two bones simultaneously, as the talar dome heightens and the posterior process diminishes, the calcaneus shortens, and the posterior facet’s slope increases (Figure 2). Additionally, as the talar dome heightens, the navicular and cuboid move inferiorly with minimal rotation about the anterior-posterior axis. The third PCA mode primarily varies with the anteromedial facet moving anterior to posterior throughout the SDs. There is minor talar dome variation but little else of note within this general multi-domain approach (Figure 2).

#### 3.1.2 Multi-level analysis in alignment shape space (alignment variant)

**FIGURE 3:**
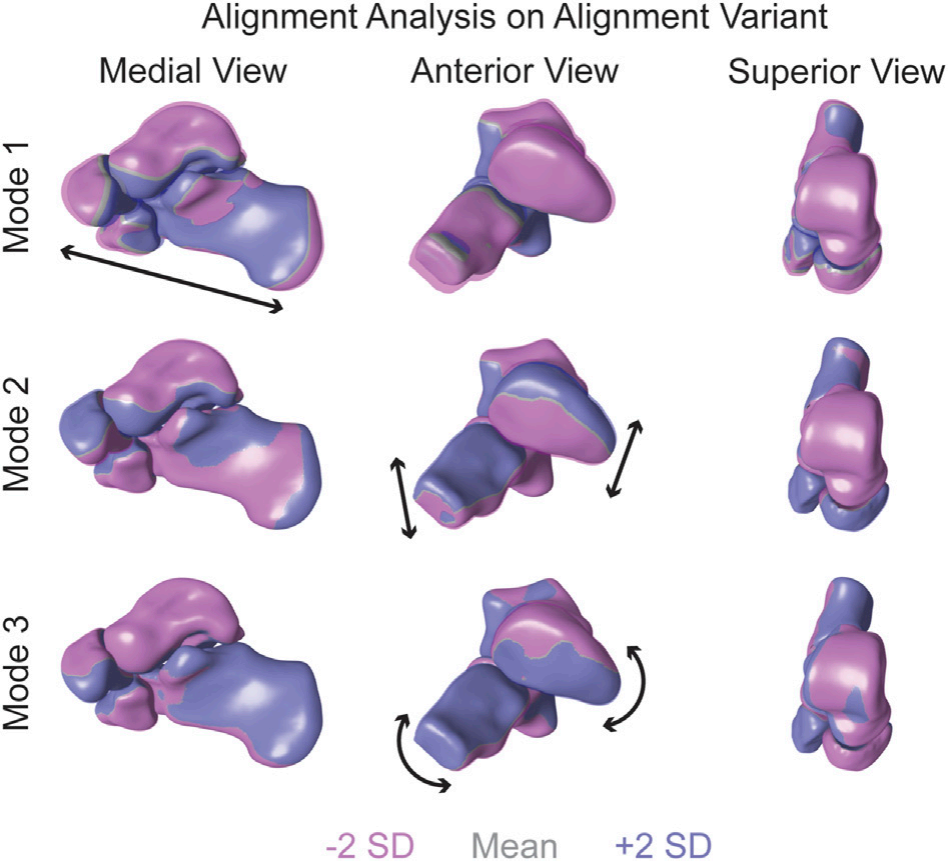
Alignment variant modes of variation one to three showing significant alignment variations at ± 2 standard deviations from the mean shape. Black arrows highlight key anatomical feature variations. Dark purple regions are +2 standard deviations and pink regions are -2 standard deviations from the mean shape in gray.

When focusing on the alignment variant, notable anatomical alignment variations were observed. The first PCA mode of variation shows an overall outward and inward movement between the bones, effectively increasing and decreasing joint space distance (Figure 3). The second PCA mode primarily highlights the superior and inferior motion of the four bones. Specifically, as the talus moves superiorly, the calcaneus, cuboid, and navicular move inferiorly (Figure 3). And the third PCA mode identifies the medial and lateral movement of the talus and calcaneus; as the talus moves medially, the calcaneus moves laterally. Additionally, as the talus moves medially, the navicular rotates superior and lateral, and the cuboid rotates inferior and medial (Figure 3).

#### 3.1.3 Multi-level analysis in morphology shape space (morphology variant)

**FIGURE 4:**
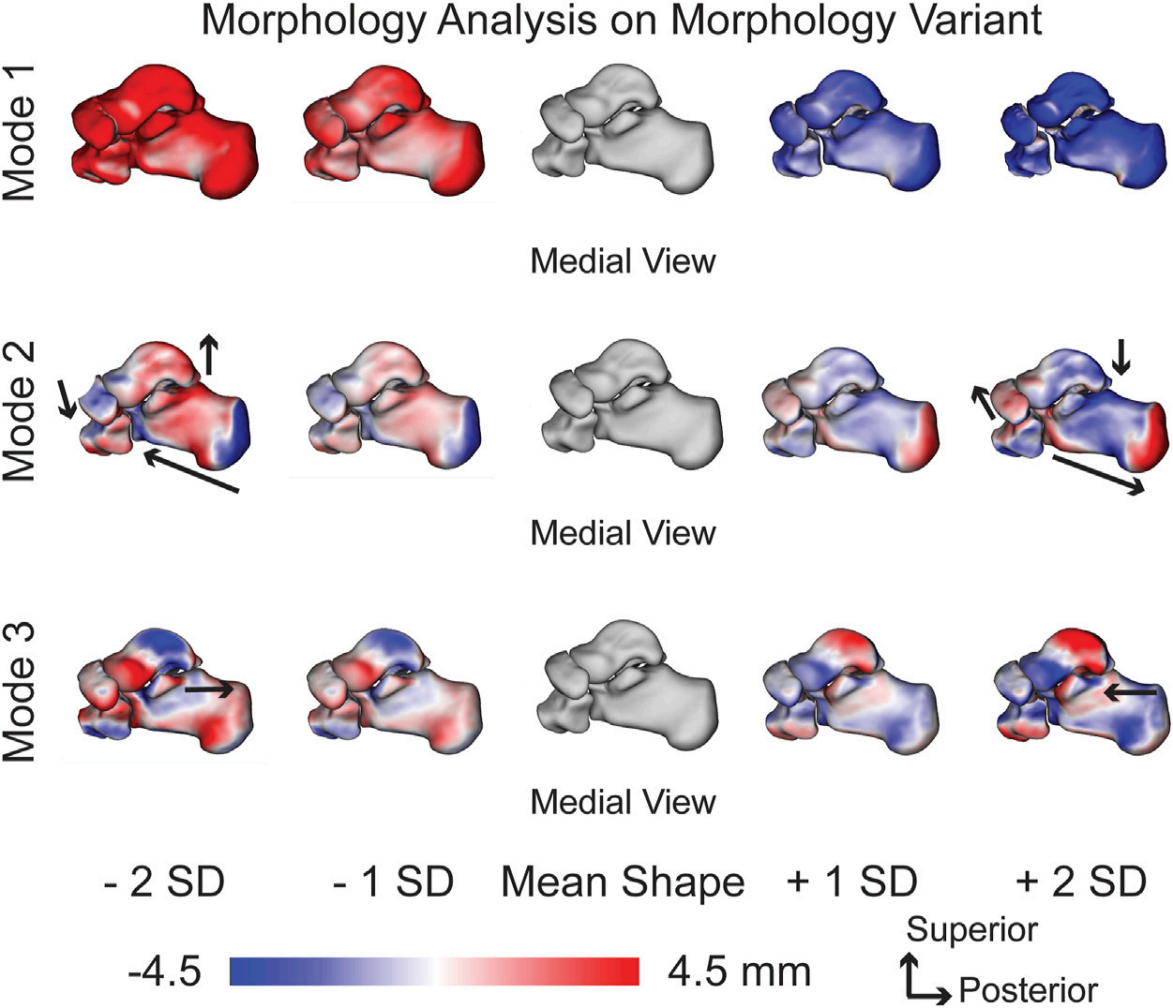
Morphology variant modes of variation one to three showing significant morphology variations at ± 1 and 2 standard deviations from the mean shape. Black arrows highlight key anatomical feature variations. Red regions are larger than the mean shape and blue regions are smaller than the mean shape with a scale in millimeters (mm).

With just observing the morphological variant, the first PCA mode of variation shows each bone growing and shrinking individually (Figure 4). The second PCA mode of variation primarily has the lengthening of the calcaneus with a decreasing posterior facet slope. There are still slight talar dome changes and slight navicular and cuboid changes, but they are not as prominent as the general multi-domain approach (Figure 4). The third PCA mode shows a similar anterior/posterior anteromedial facet variation; however, it illustrates more of a rotational component. The anteromedial facet’s slope changes throughout the SDs from a steep slope to a more flattened slope (Figure 4).

### 3.2 Joint measurements

#### 3.2.1 Coverage

##### 3.2.1.1 Subtalar joint

The coverage area for the subtalar joint was calculated for the entire subtalar joint and the posterior and anteromedial facets. The coverage area across the population for the entire joint averaged 1,519.34 ± 193.22 mm^2^ for the talus and 1,435.99 ± 171.36 mm^2^ for the calcaneus. None of the modes of variation within the MD SSM, morphology variant, or alignment variant were significantly different from the population. Moreover, the talus had a consistently larger coverage area than the calcaneus (Table 1).

**TABLE 1:** Average coverage surface area ± standard deviation (SD) for the subtalar joint (split between the posterior and anteromedial facet), talonavicular joint, and calcaneocuboid joint on both involved surfaces. *p*-values are reported from each mode and variant to the population, and *p*-values reported between each bone within each joint. Bolded *p*-values are statistically significant to the population and between each bone, respectively.

	Joint Coverage (mm^2^)															
	Subtalar			Subtalar Posterior Facet			Subtalar Anteromedial Facet				Talonavicular			Calcaneocuboid		
	Talus	Calcaneus	*p*-Value	Talus	Calcaneus	*p*-Value	Talus		Calcaneus	*p*-Value	Talus	Navicular	*p*-Value	Calcaneus	Cuboid	*p*-Value
Population	1519.34	1435.99	0.0995	805.39	862.60	0.0867	365.28		384.33	0.3636	471.22	541.36	**0.0007**	393.92c	406.46	0.4015
	± 193.22	± 171.36		± 112.42	± 127.85		± 74.76		± 77.94		± 67.08	± 75.05		55.07	± 53.86	
MD SSM Mode 1	1474.63	1377.59	0.0235	765.62	869.49	**0.0001**	340.84		329.94	0.3392	439.42	527.94	**0.0001**	368.92	383.57	0.1211
	± 168.08	± 135.69		± 54.76	± 39.55		± 33.25		± 48.39		± 52.03	± 55.15		± 33.06	± 35.21	
*p*-Value	0.3685	0.1710		0.1044	0.7901		0.1267		**0.0033**		0.0570	0.4574		**0.04**83	0.0702	
MD SSM Mode 2	1471.97	1374.17	**0.0001**	767.25	860.47	**0.0001**	340.92		338.70	0.7088	437.12	526.96	**0.0001**	368.09	380.56	**0.0001**
	± 15.15	± 19.35		± 5.58	± 28.98		± 0.99		± 30.70		± 4.33	± 2.82		± 0.83	± 7.05	
*p*-Value	0.2097	0.0682		0.0842	0.9330		0.0964		**0.0066**		0.0110	0.3237		**0.0089**	**0.0165**	
MD SSM Mode 3	1472.02	1370.44	**0.0001**	765.82	866.81	**0.0001**	336.23		324.99	**0.0001**	438.29	526.68	**0.0001**	365.49	380.44	**0.0001**
	± 53.63	± 38.34		± 64.85	± 63.68		± 5.97		± 12.30		± 3.37	± 7.13		± 16.75	± 7.96	
*p*-Value	0.2256	0.0578		0.1192	0.8789		0.0769		**0.0003**		0.0138	0.3163		**0.0132**	**0.0163**	
Alignment Variant	1468.19	1371.21	**0.0001**	767.49	869.14	**0.0001**	342.85		321.93	0.1095	440.07	526.87	**0.0001**	368.03	382.79	**0.0126**
Mode 1	± 33.88	± 22 44		± 48.18	± 35.90		± 56.53		± 35.50		± 45.32	± 4.50		± 20.50	± 21.44	
*p*-Value	0.1813	0.0569		0.1134	0.7990		0.2193		**0.0004**		0.0508	0.3213		**0.0261**	**0.0387**	
Alignment Variant	1471.55	1369.96	**0.0001**	765.48	864.13	**0.0001**	341.21		328.89	**0.0006**	436.97	526.51	**0.0001**	165.15	380.30	**0.0001**
Mode 2	± 5.24	± 1.97		± 16.84	± 25.28		± 11.56		± 13.17		± 8.27	± 0.30		± 2.86	± 2.67	
*p*-Value	0.2046	0.0505		0.0739	0.9516		0.1043		**0.0006**		0.0111	0.3086		**0.0091**	**0.0147**	
Alignment Variant	1471.21	1370.27	**0.0001**	765.42	872.61	**0.0001**	341.10		321.44	**0.0001**	436.38	526.55	**0.0001**	364.54	380.27	**0.0001**
Mode 3	± 1.04	± 1.05		± 2.44	± 9.88		± 3.07		± 7.11		± 1.24	± 1.28		± 3.03	± 2.50	
*p*-Value	0.2013	0.0515		0.0704	0.6867		0.0991		**0.0001**		0.0094	0.3100		**0.0078**	**0.0147**	
Morphology	1470.33	1381.86	0.0755	770.51	863.70	**0.0086**	342.50		341.38	0.9595	443.62	528.10	**0.0004**	371.68	386.73	0.3726
Variant Mode 1	± 196.84	± 159.62		± 148.24	± 97.37		± 89.03		± 71.42		± 99.22	± 60.69		± 55.72	± 56.18	
*p*-Value	0.3601	0.2352		0.3345	0.9712		0.3133		0.0 I%		0.2366	0.4785		**0.1462**	0.1935	
Morphology	1470.23	1373.01	**0.0001**	766.01	847.47	**0.0001**	340.39		349.92	0.3863	436.58	527.26	**0.0001**	364.65	380.65	**0.0001**
Variant Mode 2	± 31.80	± 28.77		± 48.30	± 75.36		± 1.76		± 56.65		± 12.19	± 7.04		± 0.60	± 2.13	
*p*-Value	0.1983	0.0652		0.5985	0.5985		0.0897		0.0692		**0.0109**	0.3356		**0.0079**	**0.0161**	
Morphology	1470.95	1373.02	**0.0001**	766.22	866.99	**0.0001**	338.35		326.76	**0.0042**	438.57	527.12	**0.0001**	365.55	380.65	**0.0001**
Variant Mode 3	± 35.16	± 20.83		± 63.87	± 43.78		± 9.09		± 17.97		± 4.82	± 3.51		± 17.26	± 7.55	
*p*-Value	0.2061	0.0636		0.1215	0.8666		0.0688		**0.0005**		**0.0147**	0.3293		**0.0136**	**0.0170**	

The coverage area across the population for the posterior facet averaged 805.39 ± 112.42 mm^2^ for the talus and 862.60 ± 127.85 mm^2^ for the calcaneus. Similarly to the entire subtalar joint, none of the modes of variation within the MD SSM, morphology variant, or alignment variant differed significantly from the population. However, unlike the entire subtalar joint, the calcaneus had a consistently larger coverage area than the talus (Table 1).

The coverage area across the population for the anteromedial facet averaged 365.28 ± 74.76 mm^2^ for the talus and 384.33 ± 77.94 mm^2^ for the calcaneus. While none of the modes of variation within the MD SSM, morphology variant, or alignment variant for the talus were significantly different from the population, most of them were different when comparing the calcaneus to the population. Additionally, the talus coverage area is generally larger compared to the calcaneus coverage area, even though that is not true for the population (Table 1).

##### 3.2.1.2 Talonavicular joint

The coverage area across the population for the talonavicular joint averaged 471.22 ± 67.08 mm^2^ for the talus and 541.36 ± 55.15 mm^2^ for the navicular. Mode one for the MD SSM, morphology variant and alignment variant was the only mode that was significantly similar to the population for the talar coverage area, each with *p*-values greater than 0.05. None of the *p*-values showed significant differences for the MD SSM, morphology variant or alignment variant compared to the population (Table 1). Additionally, the navicular had a significantly larger coverage area when compared to the talus in every analysis, including the population.

##### 3.2.1.3 Calcaneocuboid joint

The coverage area across the population for the calcaneocuboid joint average 393.92 ± 55.07 mm^2^ for the calcaneus and 406.46 ± 53.86 mm^2^ for the cuboid. For the calcaneus, only the first mode of the morphology variant was significantly similar compared to the population. For the cuboid, only the first modes of the MD SSM and morphology variant were significantly similar compared to the population. When comparing the coverage area of the calcaneus to the cuboid, most were significantly different (Table 1). Still, the population, MD SSM mode one, and all modes in the morphology variant were not significantly different between the bones. However, the cuboid had a larger coverage area when compared to the calcaneus for every analysis, including the population.

#### 3.2.2 Joint space distance

##### 3.2.2.1 Subtalar joint

**FIGURE 5:**
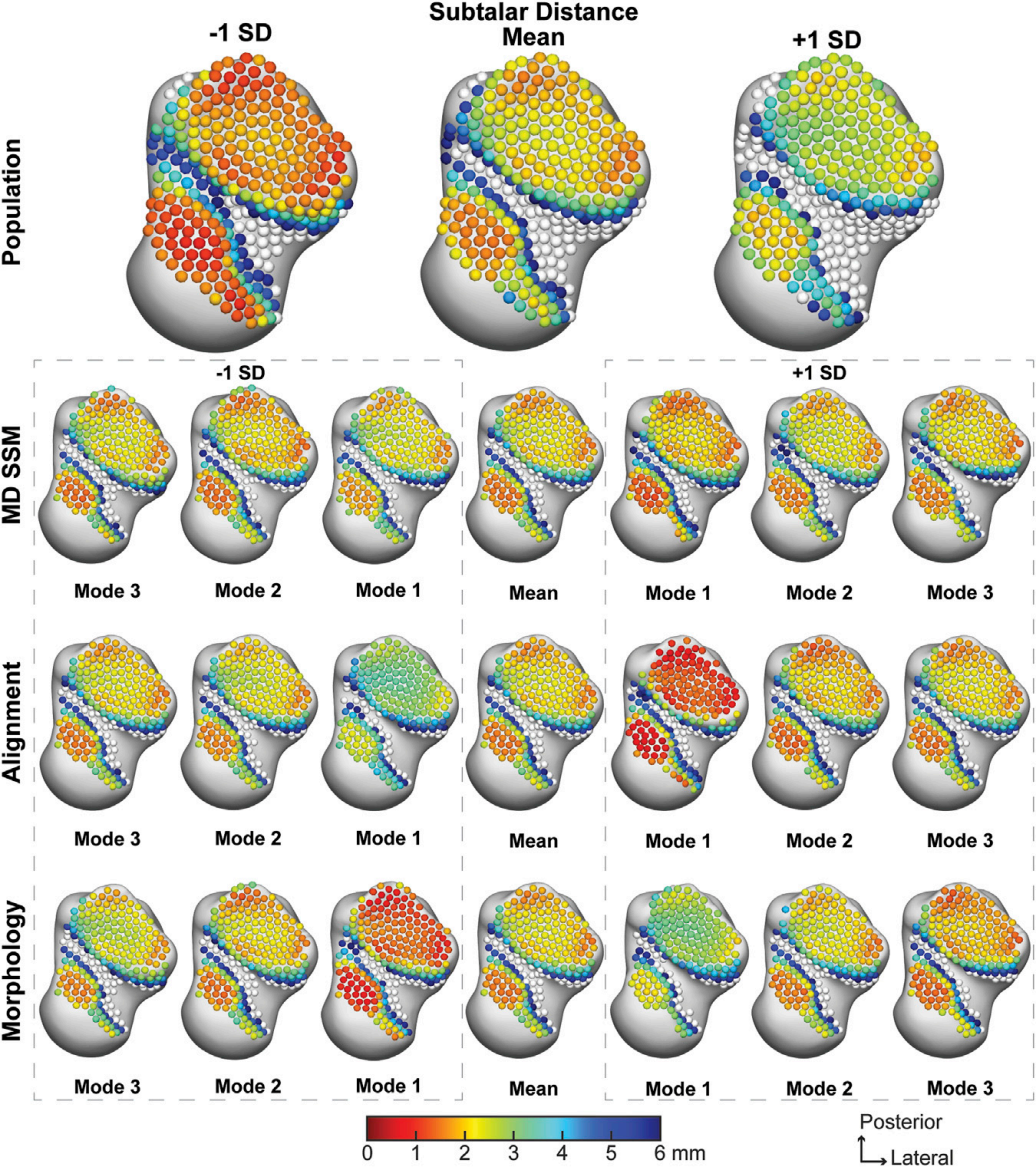
Average joint space distance with ±1 standard deviation for the subtalar articular region. Results are visualized for the population, MD SSM modes one to three, alignment variant modes one to three and morphology variant modes one to three *via* correspondence particles. Results are reported in millimeters (mm). Values larger than 6 mm in joint space distance are colored white.

The population across the entire subtalar joint averaged a joint space distance of 3.33 ± 2.06 mm, with a narrower joint space in the posterior and lateral regions of the posterior facet and the medial region of the anteromedial facet (Figure 5). The average joint space distance throughout the first three modes of the MD SSM, alignment variant and morphology variant ranged from 3.40 ± 2.10 mm to 3.54 ± 2.12 mm (Table 2). There were minor variations across the joints throughout the second and third modes of variation. And while the first mode of variation had large changes across the first SDs, none of the subtalar distance values had a *p*-value lower than 0.7109 compared to the population (Table 2).

**TABLE 2:** Average joint space distance ±standard deviation (SD) for the subtalar joint, talonavicular joint, and calcaneocuboid join. *p*-values are reported from each mode and variant to the population. Bolded *p*-values are statistically significant to the population.

Joint distance (mm)	Subtalar	Talonavicular	Calcaneocuboid
Population	3.33 ± 2.06	1.32 ± 0.43	1.67 ± 0.62
MD SSM Mode 1	3.51 ± 2.07	1.33 ± 0.21	1.65 ± 0.38
*p*-value	0.7500	0.9140	0.8869
MD SSM Mode 2	3.52 ± 2.07	1.32 ± 0.24	1.64 ± 0.38
*p*-value	0.7367	1.0000	0.8311
MD SSM Mode 3	3.48 ± 2.05	1.31 ± 0.19	1.64 ± 0.37
*p*-value	0.7896	0.9124	0.8299
Alignment Variant Mode 1	3.54 ± 2.12	1.35 ± 0.88	1.73 ± 1.13
*p*-value	0.7135	0.8742	0.8098
Alignment Variant Mode 2	3.54 ± 2.08	1.32 ± 0.30	1.64 ± 0.38
*p*-value	0.7109	1.0000	0.8311
Alignment Variant Mode 3	3.50 ± 2.10	1.32 ± 0.19	1.64 ± 0.39
*p*-value	0.7652	1.0000	0.8323
Morphology Variant Mode 1	3.40 ± 2.10	1.27 ± 0.83	1.70 ± 1.10
*p*-value	0.9021	0.7822	0.9022
Morphology Variant Mode 2	3.50 ± 2.10	1.31 ± 0.32	1.64 ± 0.39
*p*-value	0.7652	0.9231	0.8323
Morphology Variant Mode 3	3.50 ± 2.00	1.32 ± 0.21	1.65 ± 0.38
*p*-value	0.7596	1.0000	0.8869

##### 3.2.2.2 Talonavicular joint

**FIGURE 6:**
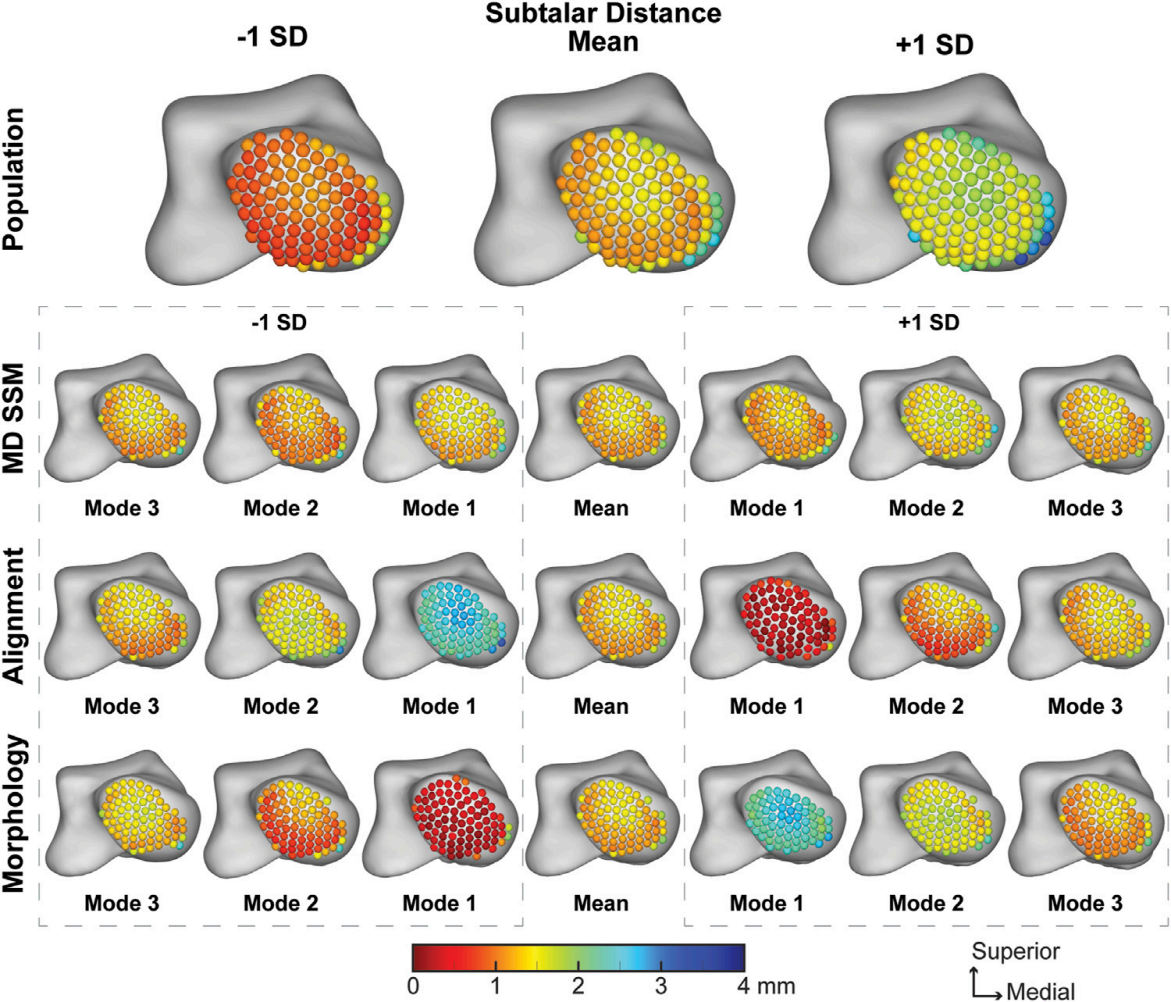
Average joint space distance with ±1 standard deviation for the talonavicular articular region. Results are visualized for the population, MD SSM modes one to three, alignment variant modes one to three and morphology variant modes one to three *via* correspondence particles. Results are reported in millimeters (mm). Values larger than 6 mm in joint space distance are colored white.

The average talonavicular joint space distance for the population was 1.32 ± 0.43 mm, with a slight widening of the medial and central aspects of the joint (Figure 6). The average joint space distance throughout the first three modes of the MD SSM, alignment variant and morphology variant ranged from 1.27 ± 0.83 mm to 1.35 ± 0.88 mm (Table 2). While the first mode expectedly had larger changes across the first SD, the second mode also had substantial variation in both morphology and alignment variants. However, they all had a similar pattern of widening distance towards the central aspect of the joint, and none had a *p*-value lower than 0.7822 compared to the population (Table 2).

##### 3.2.2.3 Calcaneocuboid joint

**FIGURE 7:**
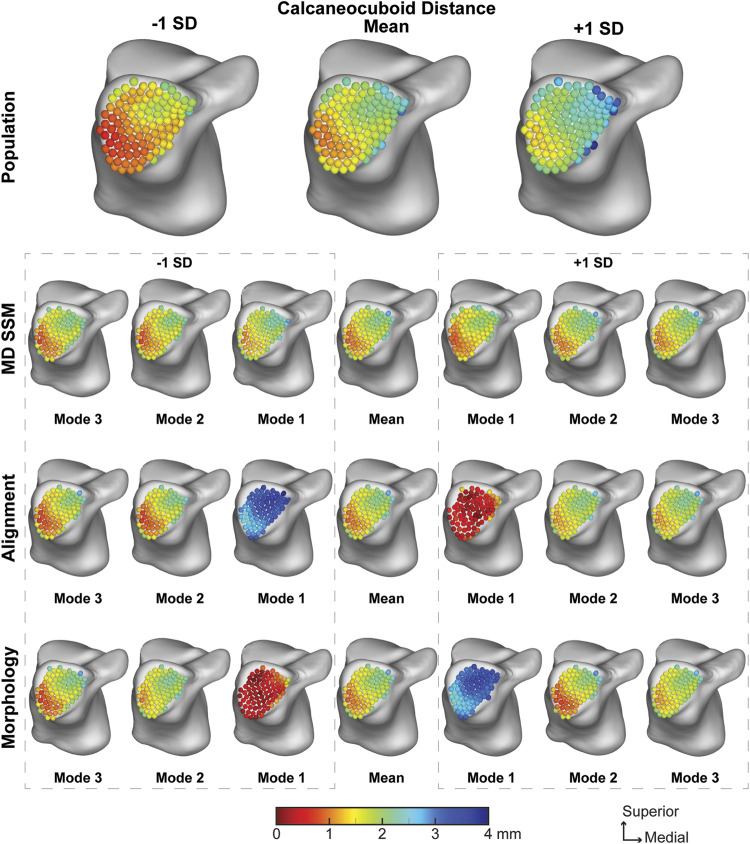
Average joint space distance with ±1 standard deviation for the calcaneocuboid articular region. Results are visualized for the population, MD SSM modes one to three, alignment variant modes one to three and morphology variant modes one to three *via* correspondence particles. Results are reported in millimeters (mm). Values larger than 6 mm in joint space distance are colored white.

The population across the calcaneocuboid joint averaged a joint space distance of 1.67 ± 0.62 mm with a narrowing in the inferolateral region of the joint (Figure 7). The average joint space distance throughout the first three modes of the MD SSM, alignment variant and morphology variant ranged from 1.64 ± 0.37 mm to 1.73 ± 1.13 mm (Table 2). Similar to the previous joints, the first mode has the widest variation across the SD, with modes two and three showing a similar pattern as the mean. Additionally, none of the calcaneocuboid distance values had a *p*-value lower than 0.8098 compared to the population (Table 2).

#### 3.2.3 Joint congruence index

##### 3.2.3.1 Subtalar joint

**FIGURE 8:**
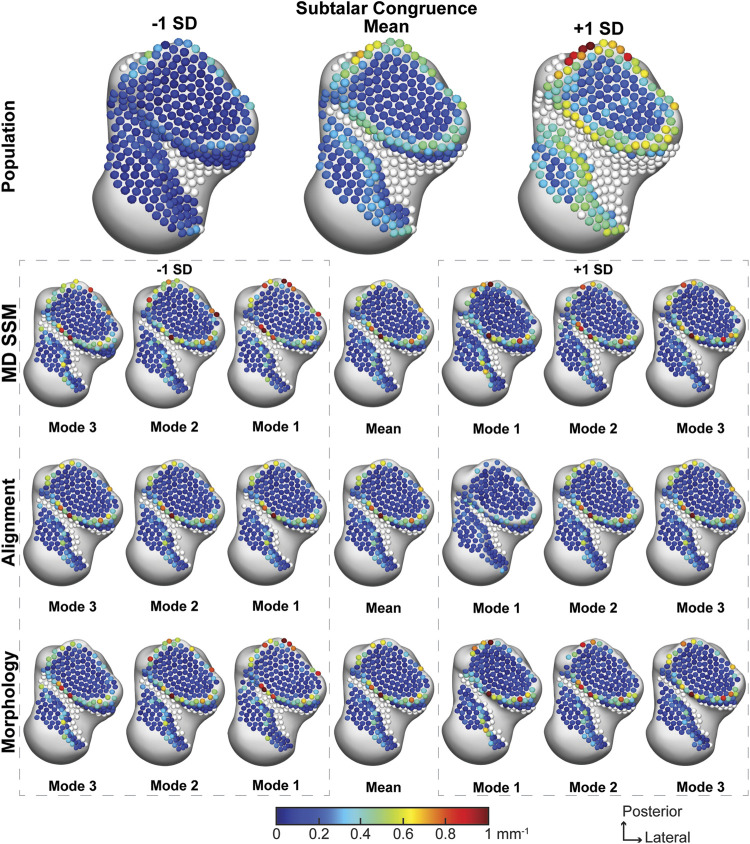
Average joint congruence indices with ±1 standard deviation for the subtalar articular region. Results are visualized for the population, MD SSM modes one to three, alignment variant modes one to three and morphology variant modes one to three *via* correspondence particles. Results are reported in inverse millimeters (mm^−1^). Values larger than 6 mm in joint space distance are colored white.

The population across the entire subtalar joint had an average congruence index of 0.24 ± 0.16 mm^−1^. With a worsening congruence index on the outer edge of the joint (Figure 8). The average joint congruence index throughout the first three modes of the MD SSM, alignment variant and morphology variant ranged from 0.16 ± 0.14 mm^−1^ to 0.20 ± 0.18 mm^−1^ (Table 2). All modes and variants had similar congruence patterns and none had a *p*-value lower than 0.0559 compared to the population (Table 3). Overall, the population congruence was consistently higher than the MD SSM, morphology variant and alignment variant.

**TABLE 3:** Average congruence index ±standard deviation (SD) for the subtalar joint, talonavicular joint, and calcaneocuboid join. *p*-values are reported from each mode and variant to the population. Bolded *p*-values are statistically significant to the population.

Congruence index (mm^−1^)	Subtalar	Talonavicular	Calcaneocuboid
Population	0.24 ± 0.16	0.23 ± 0.12	0.30 ± 0.18
MD SSM Mode 1	0.20 ± 0.18	0.16 ± 0.06	0.22 ± 0.15
*p*-value	0.3921	**0.0091**	0.0819
MD SSM Mode 2	0.18 ± 0.16	0.17 ± 0.06	0.21 ± 0.15
*p*-value	0.1742	**0.0241**	0.0512
MD SSM Mode 3	0.20 ± 0.16	0.16 ± 0.06	0.21 ± 0.14
*p*-value	0.3626	**0.0091**	**0.0453**
Alignment Variant Mode 1	0.16 ± 0.14	0.17 ± 0.06	0.21 ± 0.12
*p*-value	0.0559	**0.0241**	**0.0353**
Alignment Variant Mode 2	0.20 ± 0.16	0.16 ± 0.06	0.21 ± 0.14
*p*-value	0.3626	**0.0091**	**0.0453**
Alignment Variant Mode 3	0.20 ± 0.16	0.16 ± 0.06	0.21 ± 0.14
*p*-value	0.3626	**0.0091**	**0.0453**
Morphology Variant Mode 1	0.20 ± 0.18	0.22 ± 0.07	0.22 ± 0.15
*p*-value	0.3921	0.7099	0.0819
Morphology Variant Mode 2	0.20 ± 0.16	0.17 ± 0.06	0.21 ± 0.14
*p*-value	0.3626	**0.0241**	**0.0453**
Morphology Variant Mode 3	0.20 ± 0.16	0.16 ± 0.06	0.21 ± 0.14
*p*-value	0.3626	**0.0091**	**0.0453**

##### 3.2.3.2 Talonavicular joint

**FIGURE 9:**
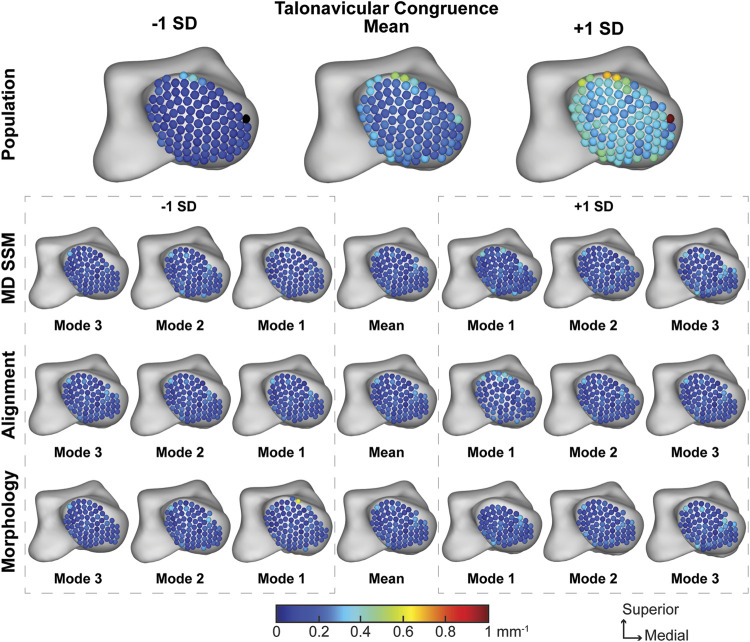
Average joint congruence indices with ±1 standard deviation for the talonavicular articular region. Results are visualized for the population, MD SSM modes one to three, alignment variant modes one to three and morphology variant modes one to three *via* correspondence particles. Results are reported in inverse millimeters (mm^−1^). Values larger than 6 mm in joint space distance are colored white.

The average talonavicular joint congruence index across the population was 0.23 ± 0.12 mm^−1,^ with subjectively consistent congruence across the joint (Figure 9). The average joint congruence index throughout the first three modes of the MD SSM, alignment variant and morphology variant ranged from 0.16 ± 0.06 mm^−1^ to 0.22 ± 0.07 mm^−1^ (Table 3). While all the modes and variants had similar congruence patterns, the only variant with a *p*-value larger than 0.05 was the first mode in the morphology variant (0.7099). All other congruence values were significantly different in a more congruent fashion compared to the population. Overall, the population congruence was consistently higher than the MD SSM, morphology variant and alignment variant.

##### 3.2.3.3 Calcaneocuboid joint

**FIGURE 10:**
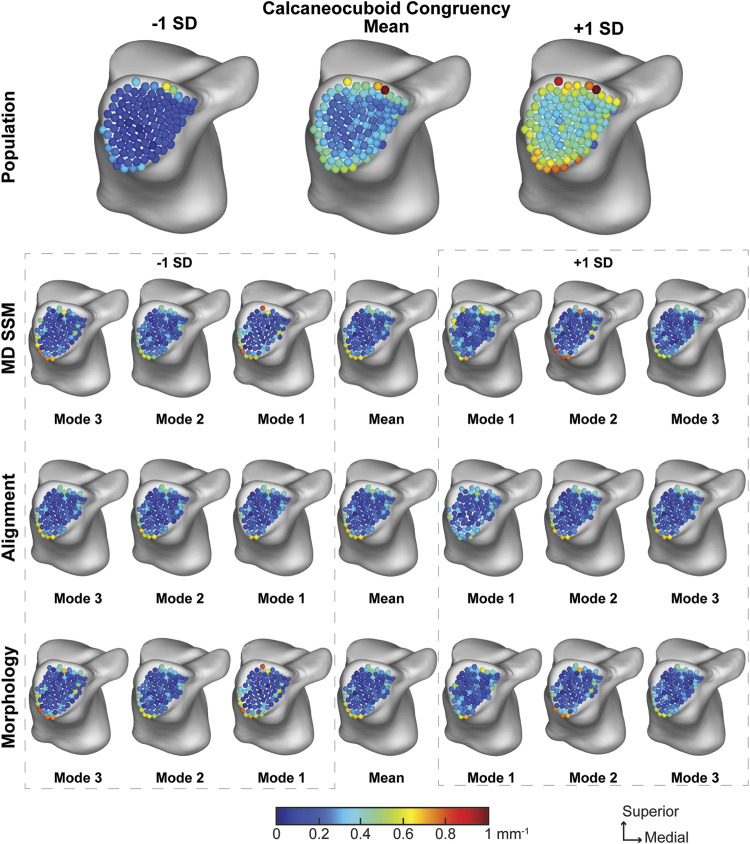
Average joint congruence indices with ±1 standard deviation for the calcaneocuboid articular region. Results are visualized for the population, MD SSM modes one to three, alignment variant modes one to three and morphology variant modes one to three *via* correspondence particles. Results are reported in inverse millimeters (mm^−1^). Values larger than 6 mm in joint space distance are colored white.

The average calcaneocuboid joint congruence index across the population was 0.30 ± 0.18 mm^−1^ with worsening congruence towards the edge of the joint (Figure 10). The average joint congruence index throughout the first three modes of the MD SSM, alignment variant and morphology variant ranged from 0.21 ± 0.12 mm^−1^ to 0.22 ± 0.15 mm^−1^ (Table 3). Only the first and second MD SSM modes and the first alignment variant mode had a *p*-value larger than 0.05. All other congruence values were significantly different in a more congruent fashion compared to the population. Overall, the population congruence was consistently higher than the MD SSM, morphology variant and alignment variant.

## 4 Discussion

The study’s primary aim was to use multi-domain SSM to characterize asymptomatic joint-level morphology and alignment variations throughout a population and determine joint-level measurements within the subtalar, talonavicular, and calcaneocuboid joints. Using those population-based joint-level measurements, we also aimed to determine if SSM can predict joint-level measurements. Moreover, we aimed to determine the validity of the multi-domain mean shapes compared to the single-domain mean shapes. The most relevant findings include: I) joint space distance is the only joint-level measurement statistically similar across all modes of variation for the MD SSM, morphology variant and alignment variant; II) the first mode in the morphology variant is statistically similar to the population across all joint-level measurements except the coverage area on the calcaneal anteromedial facet; III) MD SSM does preserve the mean shape when compared to the SD SSM mean shape.

Our MD SSM shape variations finding agree with the limited studies performed on the talus, calcaneus, navicular and cuboid. We found that the primary anatomical morphological variations included the talar trochlea height, calcaneal lengthening, and minimal variation throughout the navicular and cuboid (Melinska et al., 2015; Melinska et al., 2017; Tümer et al., 2019a; Krähenbühl et al., 2020; Lenz et al., 2021). However, we also found the relationship between the talar trochlea and calcaneal length to be interrelated. Additionally, the novel multi-level approach allowed for the separation of alignment from morphology. This approach provides insight of the talonavicular and calcaneocuboid alignment variations. Primary alignment variation occurred between the talus, navicular and cuboid. Specifically, when the talus moves inferiorly, the navicular and cuboid move superiorly and *vice versa*. But when the talus moves medially or laterally, the navicular and cuboid rotate about the anterior-posterior axis. Multiple studies have attempted to analyze and understand the role these midfoot joints have on human locomotion but have reached a wide range of conclusions. Some studies have found the talonavicular and calcaneocuboid joints to rotate throughout gait creating what is known as a locking and unlocking mechanism based on the axes of the transverse tarsal joint in one plane (Elftman, 1960; Suckel et al., 2008; Sarrafian, 2011); yet others have determined the motion within these joints did not significantly increase during gait (Blackwood et al., 2005). Moreover, other studies have determined that the plantar fascia has more of a role *via* the windlass mechanism in producing movement during gait (Hicks, 1954; Welte et al., 2021) and that there was not any observed midtarsal movement during gait (Bruening et al., 2018). There is still much to be understood about these different mechanisms and the role that the hind- and mid-foot joints have during gait, but multi-domain models may be a step towards understanding how morphology and alignment during static positioning influence these well-debated topics clinically.

Few studies have performed weightbearing joint space distance calculations of these three joints. One study that has calculated the asymptomatic subtalar joint distance for the joint as a whole reported 3.29 ± 0.87 mm compared to our measurement of 3.33 ± 2.06 mm, and when compared determined to not be statistically different from one another (*p* = 0.95) (Dibbern et al., 2021). While a small number of studies have calculated talonavicular and calcaneocuboid joint space distance, the most comparable to this study also used WBCT scans (Bernasconi et al., 2021). Bernasconi et al. reported average talonavicular and calcaneocuboid distance measurements of 1.14 ± 0.49 mm and 1.67 ± 1.26 mm, respectively. Compared to our measurements of 1.32 ± 0.43 mm for the talonavicular and 1.67 ± 0.62 mm for the calcaneocuboid, these measurements were statistically similar to one another (talonavicular: *p* = 0.28 and calcaneocuboid: *p* = 1.0). As previously mentioned, the first relevant finding of this study was that joint space distance was the only joint-level measurement statistically similar between the population and all modes of variation for the MD SSM, morphology variant and alignment variant. This finding may indicate that MD SSM, morphology variant and alignment variant models have the potential to predict joint distance measurements within a population.

When separating alignment (alignment variant) from morphology (morphology variant) in the first mode of variation, the relationship between size and joint space can be determined. As seen in the SSM figures, as the models get larger towards -2 SDs (Figure 4), they also become further apart (Figure 3). This relationship can be further seen in the joint space distance figures (Figures 5–7) with the morphology indicating that joint space is narrowing as the bone models get larger, but the alignment indicating that they are simultaneously moving further apart. This pattern indicates that individuals with larger bones subsequently have greater joint space. While joint space has been previously correlated to sex, height and weight measurements (Goker et al., 2009), further correlation to bone size and shape in the foot and ankle may be concluded from this approach. Additionally, the second relevant finding referring to the first mode of variation in the morphology variant being generally statistically similar to the population gives potential relevance to another predictive conclusion. With the first mode of the morphology variant producing the most statistically similar joint measurement results to the population, it may indicate that this specific mode can be used to predict all three measurements within a population. It also may indicate that variation across a population of these four bones is driven far more by morphology than by alignment variation. To further support this postulate, identifying patient populations with morphological variations, such as those with asymmetric ankle osteoarthritis, those suffering from PCFD, or after fracture reduction, could help better understand the role morphology plays in disease and deformity progression.

This study is not without its limitations. Soft tissues, specifically ligaments, tendons, and articular cartilage, were not included in this study. All joint-level measurements were calculated on subchondral bone surfaces, which do not consider the effects of these soft tissues when concerning alignment and joint distance. Second, WBCT scans are dependent on the native CT resolution and therefore maximum precision is 0.4 mm when considering CT segmentations for joint measurements. However, with the error spectrum being ±1 pixel, it is still less than 1 mm, therefore we feel confident in our ability to report clinically meaningful joint measurements. Third, static imaging does not capture the articulation behavior of these joints that imaging during human locomotion could capture. Further analyses on joint-level measurements during dynamic activities should be evaluated to further understand the true relationship these joints have with one another. Fourth, this study only includes bone models from healthy asymptomatic individuals. While this was intentionally done to determine healthy variations, incorporating pathological bone models in future studies could give better context of the morphological variations found in this healthy model. Further studies relating these findings to individuals with hindfoot ankle diseases will provide more relevance to clinical actions that should be taken.

In conclusion, joint-level morphology and alignment variations can be further understood using a multi-level multi-domain SSM. This study found joint space distance measurements across all modes of variation and the first mode of the morphology variation across all primary measurements to be statistically similar to the population. This may indicate using SSM to predict joint-level measurements in specific variants is a valid approach. Additionally, multi-domain SSM does preserve mean shape compared to a single-domain SSM mean shape and can confidently be used to further multi-domain SSM studies. Further studies include expanding multi-domain modeling to subsequent joints in the foot and ankle for asymptomatic and symptomatic populations. Additionally, performing multi-domain joint-level measurements during dynamic activities is necessary to fully understand the complex relationship between these joints in the foot and ankle. Future optimization schemes could be developed where the morphology and alignment variants are coherently reflected in the particle position updates of the optimized correspondence model.

## Data Availability

The raw data supporting the conclusions of this article will be made available by the authors, without undue reservation.
